# Smart Clot: An Automated Point-of-Care Flow Assay for Quantitative Whole-Blood Platelet, Fibrin, and Thrombus Kinetics

**DOI:** 10.3390/bios16020080

**Published:** 2026-01-28

**Authors:** Alessandro Foladore, Simone Lattanzio, Ekaterina Baryshnikova, Martina Anguissola, Elisabetta Lombardi, Marco Valvasori, Roberto Vettori, Francesco Agostini, Roberto Tassan Toffola, Lidia Rota, Marco Ranucci, Mario Mazzucato

**Affiliations:** 1 Sedicidodici s.r.l., 33170 Pordenone, Italy; afoladore@1612medical.com (A.F.); slattanzio@1612medical.com (S.L.); lidiarotavender@gmail.com (L.R.); 2 Department of Cardiothoracic and Vascular Anesthesia and Intensive Care, Istituto di Ricovero e Cura a Carattere Scientifico (IRCCS) Policlinico San Donato, 20097 San Donato Milanese, Italy; ekaterina.baryshnikova@grupposandonato.it (E.B.); martina.anguissola@grupposandonato.it (M.A.); marco.ranucci@grupposandonato.it (M.R.); 3 Stem Cell Unit, Department of Research and Advanced Cancer Diagnostic, Centro di Riferimento Oncologico di Aviano (CRO) IRCCS, 33081 Aviano, Italy; elombardi@cro.it (E.L.); marco.valvasori@cro.it (M.V.); rvettori@cro.it (R.V.); fagostini@cro.it (F.A.); 4 Unit of Transfusion Medicine in Oncology, Department of Transfusion Medicine, Azienda Sanitaria Friuli Occidentale, 33170 Pordenone, Italy; roberto.tassantoffola@asfo.sanita.fvg.it

**Keywords:** smart clot, point-of-care, flow-based hemostasis, platelet–fibrin interactions, thrombin generation, integrated density, microfluidics, anticoagulants, antiplatelet therapy, cardiopulmonary bypass

## Abstract

Hemostasis depends on the coordinated interaction between platelets, coagulation factors, endothelium, and shear forces. Current point-of-care (POC) assays evaluate isolated components of haemostasis or operate under nearly static conditions, limiting their ability to reproduce physiological thrombus formation. In this study, we performed the technical validation of Smart Clot, a fully automated, microfluidic POC platform that quantifies platelet aggregation, fibrin formation, and total thrombus growth under controlled arterial shear using unmodified whole blood. Recalcified citrated blood was perfused over collagen at $\dot{\gamma }$_w_ = 300 s^−1^. Dual-channel epifluorescence microscopy acquired platelet and fibrin(ogen) signals at 1 frame per second. Integrated Density time-series were fitted with a five-parameter logistic model; first derivatives and their integrals yielded standardized pseudo-volumes for platelets, fibrin(ogen), and total thrombus. Sixty-two healthy donors established reference distributions; one-hundred-thirteen patients on antithrombotic therapy assessed pharmacodynamic sensitivity. Platelet-derived parameters were approximately normally distributed, whereas fibrin(ogen) and total thrombus values followed log-normal patterns. Anticoagulants and antiplatelet agents produced graded, mechanism-consistent inhibition of all thrombus components. Cardiopulmonary bypass samples showed profound but transient suppression of platelet and fibrin activity. Across activity ranges, multiple statistical assessments supported high analytical repeatability. Smart Clot provides rapid, reproducible, flow-aware quantification of platelet–fibrin dynamics, capturing pharmacological modulation and peri-operative impairment with high sensitivity. These results support its potential as a next-generation POC assay for physiological hemostasis assessment.

## 1. Introduction

Hemostasis is a vital physiological process that prevents excessive bleeding when blood vessels are injured and allows normal blood fluidity in basal conditions. It involves a complex series of events leading to the formation of a stable blood clot, which temporarily seals the injury and preserves circulatory integrity. Understanding these mechanisms is crucial for diagnosing and treating bleeding disorders and thrombotic conditions.

Blood clotting can be divided into primary and secondary hemostasis, which operate in a tightly coordinated manner. Primary hemostasis involves platelet adhesion, activation, and aggregation at the site of vascular injury, forming the initial platelet plug [1]. Secondary hemostasis relies on the enzymatic coagulation cascade, generating insoluble, cross-linked fibrin that stabilizes the plug and incorporates blood cells [2]. Platelets and coagulation factors act interdependently: platelets provide a catalytic surface for coagulation complex assembly, while thrombin generated in secondary hemostasis further activates platelets and amplifies clot formation [3,4].

Conventional coagulation assays, although widely used in clinical laboratories, present intrinsic limitations. Many tests are performed on separated blood fractions, require exogenous activators or inhibitors, and lack the fluid-dynamics environment characteristic of the vasculature [1,5]. This reductionist approach prevents assessment of the coordinated interplay between platelets and coagulation, leading to partial representation of the hemostatic process. As a result, current diagnostics may fail to reflect a patient’s actual hemostatic status, particularly in emergency or therapeutic monitoring scenarios [6,7,8]. Microfluidic systems replicating vascular flow have been developed as research tools to study thrombus formation in vitro. These platforms have provided valuable mechanistic insights but remain complex, manual, time-consuming, and unsuitable for point-of-care (POC) implementation [9]. Despite decades of progress, there is still no clinically available method capable of integrating platelet aggregation, fibrin formation, and thrombus stabilization under physiological flow conditions.

Point-of-care testing has emerged as a strategy to monitor coagulation at the patient’s bedside. Current POC methods are limited, focusing either on platelet adhesion/aggregation or on viscoelastic properties of clot formation, often under static conditions and with restricted clinical applicability [10,11,12,13].

The Smart Clot system is a fully automated POC device designed to overcome these limitations. It reproduces the physiological interplay between platelets, coagulation factors, and shear forces, without adding external activators or inhibitors. In a microfluidic chamber, whole blood flows over a collagen-coated surface, enabling platelet adhesion and aggregation (primary hemostasis), subsequent thrombin generation, and fibrin mesh formation (secondary hemostasis) [3,10,14]. Smart Clot thus provides a quantitative assessment of the full thrombotic process, capturing both platelet and fibrin contributions and their integration in thrombus formation under near-physiological conditions.

This work reports the technical validation of Smart Clot. Specifically, we analyzed (i) healthy blood donors to establish reference distributions and assess inter-individual variability and (ii) patients with cardiovascular disease receiving antiplatelet or anticoagulant therapy to evaluate the system’s ability to detect pharmacological modulation of hemostasis. This dual approach demonstrates both the robustness of the technology in physiological conditions and its translational potential for clinical practice.

## 2. Materials and Methods

### 2.1. Device Description and Optical System Calibration

The Smart Clot instrument (Sedicidodici s.r.l., Pordenone, Italy) consists of a computerized microscopy unit equipped with a high-precision motorized stage and integrated fluorescence illumination, coupled to a disposable single-use microfluidic cartridge. The analytical workflow is fully automated and encompasses reagent handling, flow control, temperature regulation, and image acquisition. A complete technical description of the instrument, including a figure illustrating the device layout and the microfluidic flow chamber geometry, has been reported previously [14].

Each cartridge forms a rectangular microchannel (21 mm × 2.1 mm × 0.2 mm, length × width × height) made of medical-grade silicone with a glass roof coated with type I fibrillar collagen. These dimensions were selected to reproduce a standard rectangular parallel-plate flow chamber geometry, commonly adopted for platelet adhesion and thrombus formation assays under controlled arterial shear conditions. In particular, a channel height of 0.2 mm enables stable laminar flow and physiologically relevant wall shear rates with manageable flow rates, while maintaining compatibility with wide-field quantitative epifluorescence microscopy. The channel width (≈2 mm) provides a sufficiently large observation area and reduces edge effects, whereas the length (≈20 mm) ensures an adequate region of fully developed parallel flow upstream of the imaging zone. This configuration mimics a vascular surface and provides a reproducible environment for platelet adhesion and fibrin formation under flow. Whole blood is recalcified immediately before testing and perfused at a constant wall shear rate ($\dot{\gamma }$_w_ = 300 s^−1^), representative of an average value in large arterial vessels [9,15]. The wall shear rate was calculated as

$${\dot{\gamma }}_{w}=\frac{4Q}{\pi r^{3}}$$

where *Q* is the volumetric flow rate, and *r* is the hydraulic radius of the channel.

Epifluorescence microscopy was performed with a 20× objective (RMS20X, Olympus Corporation, Tokyo, Japan) and a monochrome CMOS camera (C13752-50U, Hamamatsu Photonics, Hamamatsu, Japan), providing submicron spatial resolution. Illumination was achieved with high-stability green (Mic-LED-550A) and blue (Mic-LED-500A) LEDs (Prizmatix, Holon, Israel), supplemented by a white LED source (LSQ-00-050-2-W-24V, TMS Lite, Sungai Ara Penang, Malaysia). Excitation and emission were controlled by narrow-band filters (ET546/22x, ET480/30x, and 59004m, Chroma Technology, Bellows Falls, VT, USA).

Calibration of optical response and detector homogeneity was performed using InSpeck™ Green and Orange microspheres (Thermo Fisher Scientific, Waltham, MA, USA). These 2.5 µm polystyrene beads, comparable in size to platelets, provide defined fluorescence intensities (0–100%). Calibration ensured a linear detector response and uniform illumination across the dynamic range, as recommended for quantitative fluorescence microscopy [16,17].

### 2.2. Test Procedure and Experimental Workflow

Acid-soluble type I collagen (200 µL) was introduced into the Smart Clot cartridge, and reagents from the automation kit were loaded according to manufacturer instructions. Once inserted, the cartridge was automatically heated to 37 °C and pre-incubated for 7 min to stabilize the collagen coating on the bottom of the slide of the cartridge that contains the perfusion microchannel.

During initialization, platelets were stained with DiOC6(3) (green fluorochrome) to label platelet membranes. Autofocus was achieved using micro-engraved fiducial marks on the slide. After rinsing with saline (0.9% NaCl), CaCl_2_ (10 mM), and Alexa Fluor™ 546–conjugated fibrinogen were sequentially aspirated before blood introduction.

Upon thrombin generation, labeled fibrinogen becomes incorporated into the developing fibrin network under flow. Accordingly, fluorescence-derived parameters are referred to as “fibrin(ogen)” throughout the manuscript.

The perfusion phase lasted 7 min and 30 s, during which platelets adhered and aggregated, while fibrin formed through endogenous thrombin. Images were recorded at 1 frame/second per channel, yielding an effective 2 s temporal resolution per fluorochrome.

Dual-channel fluorescence alternated between green (platelets) and red (fibrin(ogen)) without spectral overlap. Fluorescence images were captured every second per channel, alternating excitation wavelengths to separately acquire platelet and fibrin(ogen) signals. Composite pseudo-color images (merged DiOC6(3) + Alexa 546) were obtained using ImageJ v1.54i [18].

### 2.3. Image Processing and Quantitative Analysis

Automated analysis was conducted using Smart Clot software v30.0.8 (A.S.T. Biomedical, Albino, Italy). Each image sequence was binarized using adaptive thresholding optimized for platelet and fibrin(ogen) structures. Segmented stacks were merged, small artifacts (<2 pixels) were removed, and binary masks were multiplied by the original grayscale images to restore intensity information. Each pixel represented 0.4 µm^2^, enabling visualization of individual platelets (~2 µm diameter).

For each frame, the software computed the following:

- Area (µm^2^);
- Mean Gray Value (0–255);
- Integrated Density (ID) = Area × Mean Gray Value, corresponding to a pseudo-volume of adherent/aggregated elements.

The time-series of ID was plotted from t = 0 s (Ca^2+^ addition) to 450 s. Curves were fitted with a Richards’ five-parameter logistic (5PL) model using robust regression to minimize outlier impact. For comparison, a fifth-degree polynomial model (as in [14]) was applied; goodness of fit was assessed by calculating mean Robust Sum of Squares (RSS) and ∆RSS (poly − 5PL).

Across platelet, fibrin(ogen), and total thrombus curves, the 5PL model yielded superior fit quality and smoother derivatives (see the Supplementary Materials Section, Table S1). From these fits, first derivatives (rate of thrombus growth) and numerical integrals (area under the derivative curve) were computed, representing standardized thrombus pseudo-volumes under flow [19,20]. This pipeline suppressed optical noise and enhanced the reproducibility of platelet–fibrin kinetics.

### 2.4. Reagents

- Type I collagen (Merck, Darmstadt, Germany), prepared at 1–1.5 mg/mL [21];
- Alexa Fluor™ 546–fibrinogen (Thermo Fisher Scientific, Waltham, MA, USA) used at 10 µg/mL in DPBS;
- DiOC6(3) (Thermo Fisher Scientific, Waltham, MA, USA), resuspended in DMSO [22] and used at 0.87 µM (DMSO < 1%);
- Calcium chloride (10 mM) and NaCl 0.9% were of analytical grade (Merck, Darmstadt, Germany).

### 2.5. Blood Sampling from Healthy Donors and Patients

The study was approved by the Unique Regional Friulian Ethics Committee (CEUR, CRO-2017-21) and the Territorial Ethics Committee Lombardy 1 (I.R.C.C.S. San Donato Hospital, CET 40-2023), following the Declaration of Helsinki and National Law. All patients signed the study-specific informed consent.

Healthy donors (*n* = 62, aged 18–65) had no medication for ≥3 weeks and met Italian Ministry of Health eligibility criteria for blood donation, as defined by the Italian Ministerial Decree of 2 November 2015 on quality and safety requirements for blood and blood components.

Patients (*n* = 113) with cardiovascular conditions were on stable antithrombotic therapy for >4 weeks and divided into six groups:

1. Vitamin K Antagonists (VKAs), *n* = 6—Warfarin/Acenocoumarol, INR 2.01–3.21.
2. Direct Oral Anticoagulants (DOACs), *n* = 18—Anti-Xa or anti-IIa agents, INR 1.01–1.91.
3. Acetylsalicylic Acid (ASA), *n* = 24—aspirin 100 mg/day.
4. P2Y_12_ Receptor Inhibitors (P2Y_12_ inh), *n* = 11—clopidogrel 75 mg/day.
5. Dual Antiplatelet Therapy (DAPT), *n* = 32—ASA + Clopidogrel dual therapy.
6. Extracorporeal Circulation (ECC), *n* = 22—Cardiopulmonary bypass patients. All pre-ECC patients were on low-dose aspirin (100 mg/day) for secondary prevention, and during surgery were treated with heparin 300 IU/kg followed by protamine sulfate at a 1:1 heparin-to-protamine ratio [23].

Samples were collected into 3.2% sodium citrate, discarding the first tube. Processing occurred within 2.5 h at 20–25 °C to preserve platelet function.

Each healthy donor or patient was analyzed once, corresponding to a single test per individual.

### 2.6. Repeatability of the Smart Clot Assay

Intra-assay repeatability was tested using 60 whole-blood samples grouped by activity range relative to the normal donor mean area under the first derivative curve (AUC ID):

- High (≈100%), healthy donors (*n* = 20);
- Intermediate (≈50%), patients under anticoagulant/antiplatelet therapy (*n* = 20);
- Low (<30%), patients with marked therapeutic suppression (*n* = 20).

Each sample was run in duplicate, within 1 h, under identical conditions. For each pair, the bias, absolute bias, standard deviation (SD), standard error of the mean (SEM %), and intraclass correlation coefficient (two-way random, single measures, absolute agreement; ICC(2,1)) were computed to assess precision.

Low SEM (≤10%) and ICC(2,1) ≥ 0.8 were considered evidence of excellent analytical repeatability. This statistical design ensures repeatability across the full activity range of thrombus formation, from physiological to pharmacologically inhibited states (see Section 3.2) [14,19,24].

### 2.7. Statistics

All statistical analyses were performed using GraphPad Prism 10.6.1 (GraphPad Software, San Diego, CA, USA) and Microsoft Excel 365 (Microsoft Corporation, Redmond, WA, USA). The first was employed both for data processing and for the generation of graphs. Quantitative data were first tested for normality and homogeneity of variance in order to determine the appropriate statistical tests to apply. Depending on the dataset, descriptive statistics (mean ± standard deviation or median with interquartile range, IQR) were calculated, and comparative analyses were performed using parametric or non-parametric tests as appropriate.

For Integrated Density distribution histograms (Section 3.3), bin numbers and widths were selected according to the Freedman–Diaconis rule.

## 3. Results

### 3.1. Analytical Framework and Kinetic Behavior in Healthy Donors

Smart Clot analysis of 62 healthy donors (aged 18–65 years) established the reference dataset for thrombus-formation kinetics under physiological flow. Each time-series of ID for platelets, fibrin(ogen), and total thrombus was fitted using the 5PL model, which yielded excellent fitting accuracy and smoother derivatives compared to polynomial regression (see Supplementary Materials, Table S1).

**Figure 1:**
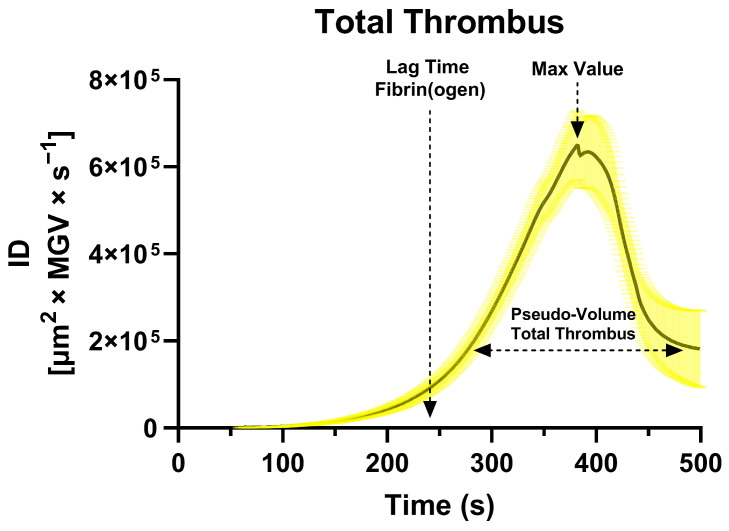
Mean first derivative curve calculated from the sigmoidal fits of 62 healthy donors’ Integrated Density (ID) time-series, representing total thrombus formation. Confidence limits (95%) are shown as shaded areas around the mean curve. The dashed arrow on the left indicates the Lag Time for fibrin(ogen) formation (~240 s), corresponding to the onset of endogenous thrombin generation. The horizontal double-headed arrow indicates the total thrombus pseudo-volume (integral of the derivative curve), while the vertical dashed arrow identifies the peak growth rate of the thrombus.

The mean first derivative curve calculated from the sigmoidal curves (total thrombus) revealed a characteristic unimodal shape (Figure 1), whose Lag Time—derived from the fibrin(ogen) derivative—occurred at approximately 241 ± 39 s, marking the onset of endogenous thrombin generation. AUC ID values quantified the thrombus pseudo-volume (platelet + fibrin(ogen) components), while the peak of the derivative represented the maximal rate of thrombus growth.

Mean and percentile distributions of AUC ID values demonstrated that platelet values were approximately normally distributed, whereas fibrin(ogen) and total thrombus values exhibited right-skewed, log-normal patterns, indicating greater variability in secondary-hemostasis dynamics (see Section 3.3). These parameters provide a comprehensive kinetic fingerprint of physiological thrombus formation in native whole blood. The peak value of the first derivative is omitted from the calculations for simplicity and pending full validation.

### 3.2. Intra-Assay Repeatability of Smart Clot

Intra-assay repeatability was evaluated across three analytical ranges of thrombus-formation activity: high (≈100% of normal donor AUC ID), intermediate (≈50%), and low (<30%) (Table 1).

Each group comprised 20 whole-blood samples tested in duplicate under identical conditions.

Across all activity levels, SEM values remained below 10%, and ICC(2,1) ranged 0.7–0.9, confirming excellent intra-assay precision. Bias and standard deviation analyses showed no systematic drift between replicates, even in samples with markedly reduced AUC ID. The method, therefore, demonstrated consistent reproducibility over a broad dynamic range of platelet and fibrin contributions.

These results validate the system’s analytical robustness as a quantitative POC platform, comparable or superior to established microfluidic and viscoelastic assays [5,9,10]. The low dispersion across biological activity gradients supports its suitability for both research and clinical monitoring contexts.

### 3.3. Distribution of Quantitative Thrombus Parameters

Blood samples from 62 healthy donors (aged 18–64 years, without stratification for sex, weight, or lifestyle) were analyzed using the Smart Clot system. Quantitative parameters were derived from the integrals of the first derivative of the fitted sigmoidal curves describing platelet aggregation, fibrin(ogen) formation, and total thrombus growth. These integrals correspond to the pseudo-volume of the thrombus expressed in AUC ID units, providing a standardized quantitative descriptor of clot kinetics and composition.

**Figure 2:**
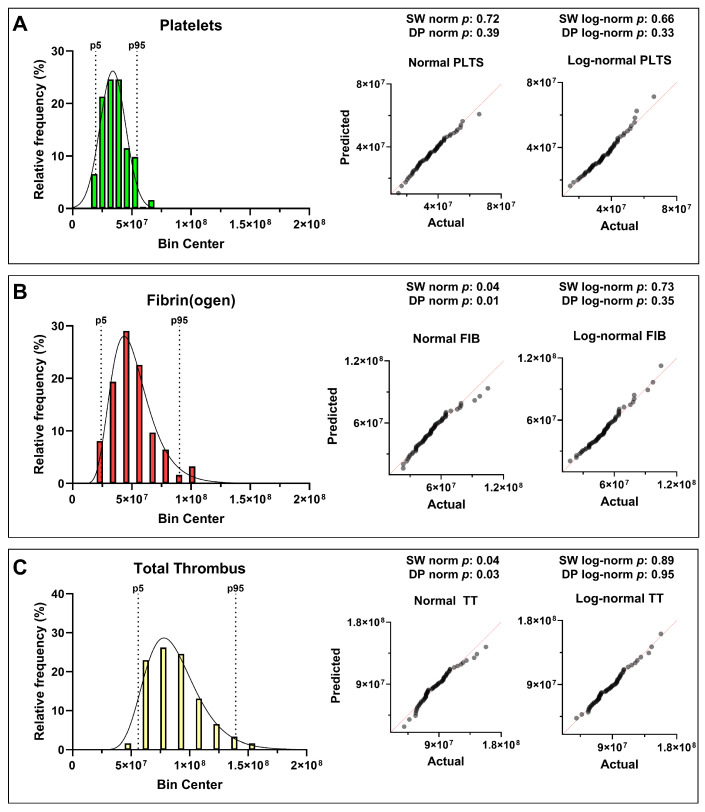
Histograms (panels’ (**left**) side) with probability density overlays and corresponding Q–Q plots (panels’ (**right**) side) show the distributions of (**A**) platelets (PLTS), (**B**) fibrin(ogen) (FIB), and (**C**) total thrombus (TT) AUC ID in 62 healthy donors. For each histogram, the 5th and 95th percentiles are indicated by dashed lines. Platelet-derived values followed an approximately normal distribution, while fibrin(ogen) and total thrombus values exhibited right-skewed log-normal behavior. Normality and log-normality were assessed by the Shapiro–Wilk (SW) and D’Agostino–Pearson (DP) tests, of which *p*-values are reported in the figure, as in Table S2.

Statistical analysis revealed distinct distribution patterns for the three hemostatic components. Platelet-derived AUC ID values followed an approximately normal (Gaussian) distribution, reflecting a relatively uniform aggregation behavior within the donor population. In contrast, fibrin(ogen)- and total thrombus-derived parameters showed a right-skewed log-normal distribution, with the shift toward higher values becoming more pronounced when platelet and fibrin(ogen) contributions were combined (Figure 2, Table 2).

The distribution of measured parameters was assessed using the Shapiro–Wilk and D’Agostino–Pearson tests (α = 0.05). Platelet data appeared approximately normally distributed, with *p* > 0.05 for both normality and log-normality tests, indicating that neither distribution could be rejected. Fibrin(ogen) and total thrombus parameters showed *p* < 0.05 for normality but *p* > 0.05 for log-normality, i.e., normality was rejected while log-normality could not be rejected, consistent with an asymmetric distribution skewed toward higher thrombus pseudo-volumes. These results suggest that, even among apparently healthy individuals, the global hemostatic response under flow tends to evolve toward increased fibrin contribution and integrated thrombus size—an intrinsic variability that may underlie population-level heterogeneity in coagulation potential. The statistical analysis results, including *p*-values and the corresponding distribution interpretation, are provided in Supplementary Table S2.

### 3.4. Effects of Anticoagulant and Antiplatelet Therapy on Thrombus Formation

Smart Clot testing revealed distinct inhibitory profiles associated with different classes of antithrombotic therapy under physiological flow conditions.

**Figure 3:**
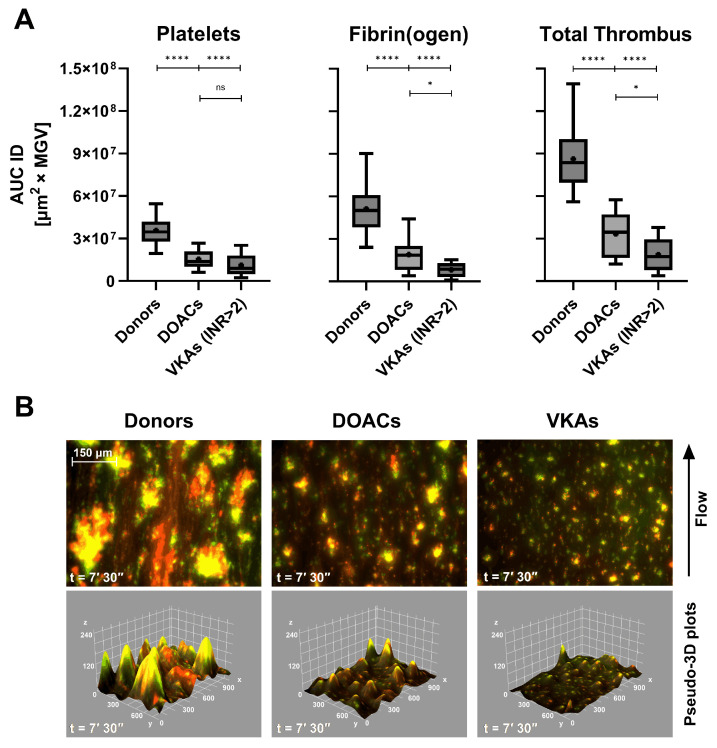
(**A**) Box-and-whiskers plots (median, mean, and 5th–95th percentiles) of platelets, fibrin(ogen), and total thrombus pseudo-volumes (AUC ID) in healthy donors (*n* = 62), patients on direct oral anticoagulants (DOACs, *n* = 18), and vitamin K antagonists (VKAs, *n* = 6, INR > 2). A consistent reduction in thrombus formation was observed, particularly in total thrombus Integrated Density (*p* < 0.0001 vs. donors). (**B**) Composite fluorescence images and pseudo-3D renderings of total thrombi (platelets in green, fibrin(ogen) in red, and merged in yellow) at 7′30″ (scale bar = 150 µm). ns: *p* > 0.05; *: *p* < 0.05; and ****: *p* < 0.0001.

In patients treated with DOACs or VKAs, both platelet- and fibrin(ogen)-derived AUC ID values were markedly reduced compared with healthy donors (Figure 3A). The decrease was most pronounced in total thrombus formation, confirming a global anticoagulant effect.

Interestingly, VKAs-treated patients with subtherapeutic INR values (1.27–1.94, data shown in Supplementary Materials, Table S3) exhibited intermediate Total Thrombus AUC ID levels (mean = 4.75 × 10^7^ µm^2^ × MGV), suggesting that Smart Clot quantitatively captures gradations of anticoagulant intensity. This finding indicates a potential correlation between thrombus pseudo-volume and pharmacodynamic effect, although the exact relationship with INR remains to be clarified.

Representative fluorescence composites (Figure 3B) confirm the quantitative data: compared with donor controls, DOACs and VKAs produced progressively smaller, less cohesive thrombi, with reduced co-localization of platelet (green) and fibrin(ogen) (red) signals and corresponding flattening of the 3D Integrated Density profiles. Quantitative AUC ID parameters are provided in Supplementary Table S3.

**Figure 4:**
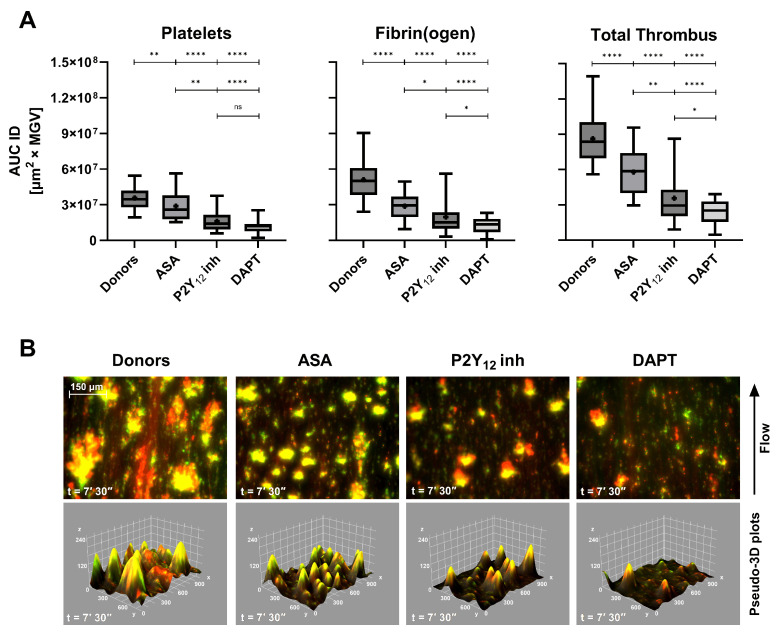
(**A**) Box-and-whiskers plots (median, mean, and 5th–95th percentiles) of platelets, fibrin(ogen), and total thrombus pseudo-volumes (AUC ID) in healthy donors (*n* = 62), patients treated with acetylsalicylic acid (ASA, *n* = 24), clopidogrel (P2Y_12_ inh, *n* = 11), and dual antiplatelet therapy (DAPT: ASA + clopidogrel, *n* = 32). All groups exhibited progressive reductions in thrombus pseudo-volume, with the strongest inhibition under DAPT (*p* < 0.0001 vs. donors). (**B**) Composite fluorescence images and pseudo-3D renderings of total thrombi (platelets in green, fibrin(ogen) in red, and merged in yellow) at 7′30″ (scale bar = 150 µm). A visual comparative video (Movie S1) illustrates thrombus development in healthy vs. DAPT samples. ns: *p* > 0.05; *: *p* < 0.05; **: *p* < 0.01; and ****: *p* < 0.0001.

Antiplatelet agents exerted a more selective inhibition pattern (Figure 4A). ASA caused a moderate reduction in total thrombus AUC ID, while clopidogrel (P2Y_12_ receptor inhibitor) induced a more marked decrease, consistent with its central role in ADP-mediated aggregation. DAPT resulted in a synergistic effect, producing the lowest total thrombus values across all treatment groups (*p* < 0.0001 vs. controls). These findings reflect the complementary mechanisms of ASA and P2Y_12_ receptor inhibitors blockade on primary hemostasis under flow. Qualitative imaging (Figure 4B) further corroborates the quantitative inhibition: the merged fluorescence images show diminished platelet–fibrin(ogen) overlap and reduced yellow co-localization, mirrored by shallow pseudo-3D reconstructions. The full-length comparative video between a healthy donor and a DAPT patient is provided in Supplementary Video S1, illustrating the markedly slowed kinetics and diminished thrombus architecture induced by dual therapy. Quantitative AUC ID parameters are provided in Supplementary Table S4.

**Figure 5:**
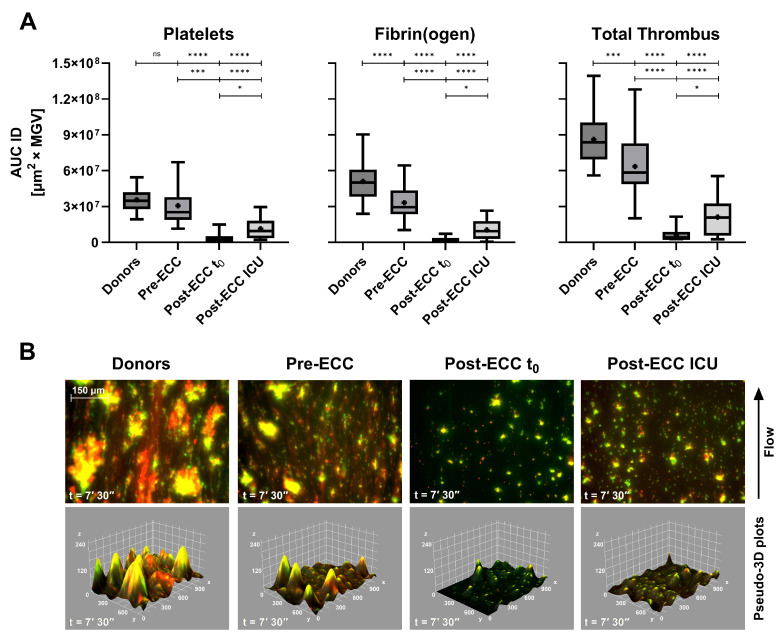
(**A**) Box-and-whiskers plots (median, mean, 5th–95th percentiles) of platelets, fibrin(ogen), and total thrombus pseudo-volumes (AUC ID) in healthy donors (*n* = 62), pre-Extracorporeal Circulation (ECC) patients (*n* = 22), post-ECC t_0_ (*n* = 6, 5–10 min after protamine 300 IU/kg), and post-ECC ICU samples (*n* = 22, ≈1.5 h after protamine). (**B**) Composite fluorescence images and pseudo-3D renderings of total thrombi (platelets in green, fibrin(ogen) in red, and merged in yellow) at 7′30″ (scale bar = 150 µm). Corresponding pseudo-3D reconstructions (Integrated Density) show marked reductions in platelet contribution and overall thrombus volume post-ECC, particularly at ICU admission. Quantitative laboratory parameters are summarized in Table 3. ns: *p* > 0.05; *: *p* < 0.05; ***: *p* < 0.001; and ****: *p* < 0.0001.

In patients undergoing cardiopulmonary bypass (ECC), Smart Clot detected profound but transient impairment of thrombus formation (Figure 5A). Post-ECC samples collected immediately after protamine administration (t_0_) exhibited minimal platelet and fibrin(ogen) activity, while samples obtained at ICU admission (≈1.5 h later) showed partial recovery. These changes paralleled hematological alterations: platelet count fell by ≈35% on average (range 17–62%), and fibrinogen levels declined from 191 to 484 mg/dL pre-ECC to 106–305 mg/dL post-ECC ICU (Table 3).

Pre-ECC samples, which represent the patients’ basal hemostatic state prior to Extracorporeal Circulation, showed a pattern comparable to that observed in ASA-treated subjects (Figure 4A). Platelet-derived Integrated Density displayed a mild, non-significant reduction relative to healthy donors, whereas fibrin(ogen)-derived values were significantly lower. This behavior is consistent with the chronic low-dose aspirin regimen (≈100 mg/day) recommended for secondary cardiovascular prevention and routinely administered in this patient population (see Section 2: Materials and Methods). Despite the marked suppression of platelet, fibrin(ogen), and total thrombus AUC IDs in both post-ECC t_0_ and post-ECC ICU samples, conventional plasma-based indices (INR and aPTT) remained within or close to normal ranges (Table 3). This divergence suggests that Smart Clot detects functional impairments of thrombus formation that are not captured by routine coagulation assays in the early post-bypass phase. The corresponding merged fluorescence images and pseudo-3D reconstructions (Figure 5B) reveal near-absence of organized thrombus immediately after ECC, followed by partial re-establishment of platelet–fibrin(ogen) co-localization during early recovery. Quantitative AUC ID parameters are provided in Supplementary Table S5.

Overall, these results demonstrate that Smart Clot effectively discriminates between the pharmacodynamic signatures of anticoagulant and antiplatelet therapies, as well as surgery-induced coagulopathy, under flow. The concordance between quantitative (AUC ID) and qualitative (imaging) findings highlights the system’s sensitivity in detecting both the magnitude and temporal evolution of thrombus inhibition.

## 4. Discussion

### 4.1. Physiological Relevance of Smart Clot Under Flow

A defining feature of the Smart Clot assay is its ability to reproduce, within a fully automated point-of-care configuration, the coordinated sequence of events that governs thrombus formation under physiological flow.

**Figure 6:**
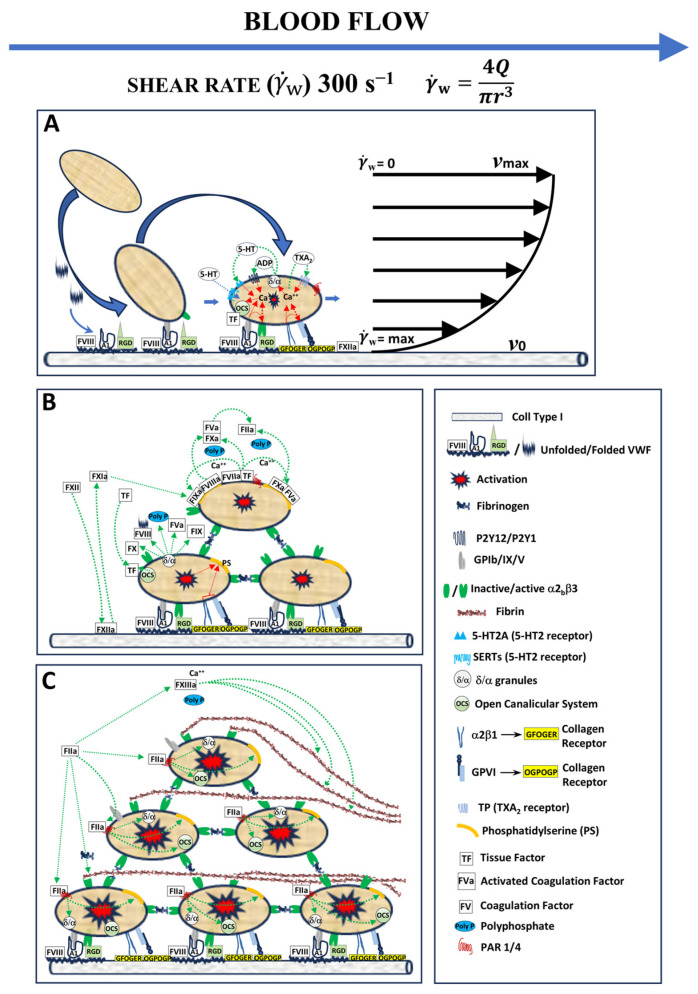
(**A**) Platelets are initially tethered to exposed collagen via von Willebrand factor (vWF) interaction with GPIb-IX-V. Firm adhesion follows through αIIbβ3–vWF, α_2_β_1_–collagen, and GPVI–collagen binding, triggering Ca^2+^ mobilization, αIIbβ3 activation, and release of ADP, serotonin, and thromboxane A_2_ (TXA_2_). These mediators amplify platelet recruitment. Activated platelets expose phosphatidylserine (PS) and tissue factor (TF) through the open canalicular system (OCS), providing catalytic surfaces for thrombin generation and linking primary and secondary hemostasis. On the right, the laminar velocity (gray) and shear rate (black) profiles illustrate how near-wall gradients ($\dot{\gamma }$w = 300 s^−1^) govern platelet transport, adhesion, and thrombus localization under physiological flow. (**B**) After adhesion, platelets aggregate through αIIbβ3 engagement and release α-granule components—fibrinogen, vWF, fibronectin, and coagulation cofactors—stabilizing the developing thrombus. Polyphosphates (polyPs) released from activated platelets enhance the assembly of the intrinsic (FIXa–FVIIIa) and extrinsic (FVIIa–TF) tenase complexes, which converge in prothrombinase (FXa–FVa) formation, catalyzing robust thrombin generation. (**C**) In the final phase, thrombin amplifies platelet activation via PAR-1 and PAR-4, and local binding to GPIb-IX-V increases its local concentration. Platelets expose PS, supporting coagulation complex assembly, while thrombin converts fibrinogen into fibrin, which polymerizes and is stabilized by FXIII-mediated cross-linking. The resulting platelet–fibrin network forms a cohesive hemostatic plug replicating the physiological environment simulated by the Smart Clot system.

Consistent with the recommendations of the Biorheology Subcommittee of the SSC of the ISTH, Smart Clot integrates native whole blood, collagen as a physiologically relevant substrate, and controlled shear flow to preserve the spatiotemporal sequence of thrombus formation [9]. Under these conditions, platelet recruitment is initiated by von Willebrand Factor (vWF)-mediated tethering through the GPIb–IX–V complex, followed by firm adhesion and activation via αIIbβ3–vWF, GPVI, and α_2_β_1_ interactions [1,2,3,25,26]. These early adhesive events trigger intracellular Ca^2+^ signaling and granule secretion. This event promotes phosphatidylserine exposure and platelet-associated tissue factor activity, which together support the assembly of procoagulant enzyme complexes on the platelet surface [2,3,4,5,27,28,29,30,31]. This integrated biomechanical and biochemical environment distinguishes Smart Clot from conventional plasma-based assays and viscoelastic systems, which primarily interrogate isolated components of coagulation or fibrin mechanics under static or artificially activated conditions. By preserving shear-dependent platelet function and endogenous thrombin generation, Smart Clot captures the functional coupling between primary and secondary hemostasis that underlies physiological thrombus growth [7,14]. The original schematic in Figure 6, grounded in the recent literature, provides the reference model through which Smart Clot-derived measurements of platelet, fibrin(ogen), and total thrombus pseudo-volumes should be read and discussed. Importantly, this design choice is not intended as a qualitative demonstration per se. Rather, it represents the prerequisite that enables the system to resolve pharmacological modulation and perioperative perturbations of thrombus formation observed in subsequent analyses.

The sensitivity of Smart Clot to antiplatelet agents, anticoagulants, and cardiopulmonary bypass–associated coagulopathy can therefore be interpreted as a direct consequence of operating within a flow-dependent, physiologically faithful hemostatic context, bridging experimental microfluidic models [11,32] and clinically applicable point-of-care diagnostics.

### 4.2. Analytical Robustness and Comparison with Conventional POC Systems

Smart Clot exhibited high analytical repeatability across the entire thrombus-formation spectrum, with SEM ≤ 10% and ICC(2,1) ≥ 0.8 for all parameters. The dynamic response was captured through sigmoidal kinetic modeling, which better described thrombus growth than fifth-degree polynomial fits (Table S1), confirming the robustness of image-based quantification [33].

Unlike viscoelastic systems (TEG and ROTEM) that mainly reflect fibrin mechanics [5,24], Smart Clot quantifies discrete and integrated components—platelet, fibrin(ogen), and total thrombus pseudo-volumes—derived directly from real-time microscopy. Platelet-specific assays (PFA-100, aggregometry) and global thrombin-generation tests (TGTs) provide partial functional readouts [34,35] and require longer analysis times. In contrast, Smart Clot combines these functional dimensions in a single automated 15 min test, capturing the interplay of shear, adhesion, enzymatic activation, and structural stabilization in whole blood.

### 4.3. Clinical Interpretability and Therapeutic Modulation

Quantitative Smart Clot parameters mirrored the expected pharmacological modulation of hemostasis. In anticoagulated patients, DOACs and therapeutic-range VKA groups showed significant reductions in platelet, fibrin(ogen), and total thrombus pseudo-volumes compared with healthy donors, confirming inhibition of both primary and secondary pathways. Interestingly, sub-therapeutic VKAs (INR 1.27–1.94; data shown in Supplementary Materials, Table S3) yielded intermediate values (mean ≈ 4.8 × 10^7^ AUC ID), demonstrating proportional sensitivity of the assay to graded anticoagulation.

Antiplatelet therapy produced a stepwise suppression of total thrombus volume: ASA > clopidogrel > dual therapy (DAPT). The accompanying pseudo-3D reconstructions (Figure 4B) visually confirmed the progressive loss of platelet–fibrin cohesion, particularly evident under DAPT. This observation is further supported by the comparative Supplementary Video S1 (control vs. DAPT). It should be noted that the greater variability observed in the clopidogrel group may be related, at least in part, to the absence of systematic screening for high on-treatment platelet reactivity using vasodilator-stimulated phosphoprotein (VASP) phosphorylation assays. This consideration is particularly relevant given the well-documented prevalence of clopidogrel non-responsiveness in otherwise stable patients [36]. Interestingly, in ASA-treated patients, the inhibitory effect appeared proportionally greater on fibrin(ogen)-derived than on platelet-derived AUC ID values. This finding suggests that low-dose aspirin may attenuate the platelet-dependent support of thrombin generation by limiting procoagulant phosphatidylserine exposure and assembly of coagulation complexes on the platelet surface, beyond its effect on bulk aggregation [37,38].

In cardiopulmonary bypass (ECC) patients, Smart Clot detected a marked reduction in thrombus formation immediately after protamine neutralization (post-ECC t_0_) and during early ICU admission, consistent with transient postoperative hypocoagulability. This correlated with reduced platelet counts and fibrinogen concentrations (Table 3), underscoring the assay’s ability to reflect multifactorial impairment of hemostasis [39].

Collectively, these results demonstrate that Smart Clot can discriminate the functional impact of major antithrombotic therapies and perioperative conditions within a single, short-duration assay, providing an integrated fingerprint of thrombus dynamics rather than isolated endpoints.

Of note, the disproportionate suppression of fibrin(ogen) versus platelet pseudo-volumes observed in ASA-treated patients suggests that, under flow, low-dose aspirin may preferentially impair the procoagulant platelet phenotype rather than simple adhesive/aggregatory function. This interpretation is consistent with evidence that a small subset of highly procoagulant, phosphatidylserine-exposing, TF-positive platelets provides the dominant membrane scaffold for tenase and prothrombinase assembly and thereby governs thrombin generation and fibrin growth under arterial shear [4,15]. In this context, COX-1 inhibition and reduced TXA_2_-dependent signaling could limit the transition of activated platelets into this fully procoagulant state, leading to a relatively stronger impact on fibrin formation than on the early phases of platelet accumulation captured by Smart Clot.

### 4.4. Limitations and Future Perspectives

This study represents a technical validation rather than a clinical trial. Sample size was limited in some subgroups—particularly post-ECC t_0_ (*n* = 6)—and anticoagulant classes (anti-Xa vs. anti-IIa) were pooled due to recruitment constraints. Moreover, ethical and protocol limitations on blood-draw volumes precluded direct statistical comparisons with other POC systems (TEG, PFA-100, and TGT).

Nevertheless, the existing literature supports the superior physiological relevance of flow-based assays [6,7,11,15]. Smart Clot uniquely combines this physiological environment with automation and quantitative imaging, positioning it as a potential next-generation tool for translational hemostasis research. Its capacity to simultaneously quantify platelet, fibrin(ogen), and total thrombus parameters suggests utility for detecting subclinical hyper- or hypocoagulability, therapy monitoring, and patient-specific risk assessment.

The log-normal distribution observed for fibrin(ogen) and total thrombus parameters in healthy donors mirrors the right-skewed population distributions reported for von Willebrand factor and factor VIII levels, which are well-recognized biochemical determinants of inter-individual thrombotic risk. This parallel suggests that the upper tail of the fibrin-driven thrombus response captured by Smart Clot may reflect a physiologic propensity toward heightened coagulation potential, even within clinically normal ranges [40,41,42].

Several studies have shown that PT/INR and aPTT have limited sensitivity to clinically relevant coagulopathies in cardiopulmonary bypass patients because they assess only isolated plasma pathways and do not reflect platelet-dependent thrombin generation or fibrin formation under flow [43,44].

In our cohort, Smart Clot identified substantial reductions in platelet contribution and fibrin-dependent thrombus formation after ECC, even when INR and aPTT remained largely normal. This discrepancy is consistent with reports indicating that early post-bypass hemostatic impairment is often multifactorial—platelet dysfunction, reduced phospholipid surface availability, hemodilution, and fibrinogen depletion—and these conditions are not reliably detected by routine clot-time assays.

These findings support the concept that whole-blood, flow-based measurements provide additional information on thrombus-forming capacity beyond conventional plasma tests, particularly in high-risk perioperative settings.

From a clinical perspective, the Smart Clot system may find practical application in several settings where a comprehensive, flow-dependent assessment of hemostasis is clinically relevant. These include acute and emergency care, cardiology and interventional cardiology, for the functional assessment of antiplatelet and anticoagulant therapies, and cardiac surgery and Extracorporeal Circulation, where complex, multifactorial coagulopathies frequently occur. Additional potential application areas include perioperative and critical care settings, as well as oncology, particularly in the context of innovative anticancer treatments such as targeted therapies and immunotherapies, where conventional coagulation assays often fail to capture treatment-associated hemostatic alterations under flow.

Future efforts will focus on prospective clinical validation in the above-mentioned complex scenarios, as well as on algorithmic expansion to extract additional parameters from video data (e.g., clot heterogeneity, fibrin network density, and spatial dynamics).

In summary, Smart Clot introduces a paradigm shift from static, reagent-driven coagulation testing to dynamic, physiology-based functional profiling. By capturing the synergy between platelets and coagulation in real time, it may fill longstanding diagnostic gaps in personalized hemostasis evaluation.

## Figures and Tables

**Table 1:** Mean bias, mean absolute bias, standard deviation (SD), standard error of the mean (SEM, % of mean), and intraclass correlation coefficient (two-way random, single measures, absolute agreement; ICC(2,1)) for platelet, fibrin(ogen), and total thrombus mean area under the first derivative curve (AUC ID) across three analytical classes: high (≈100%), intermediate (≈50%), and low (<30%) relative to normal donor means. Each group comprised 20 whole-blood samples tested in duplicate under identical conditions. ICC(2,1) ≥ 0.8 and SEM ≤ 10% indicate high repeatability.

	Platelets AUC ID			Fibrin(ogen) AUC ID			Total Thrombus AUC ID		
	≈100%	≈50%	<30%	≈100%	≈50%	<30%	≈100%	≈50%	<30%
Bias	−233.4	132.4	−40.0	−35.1	−40.0	−67.4	−268.5	92.5	−107.4
|Bias|	655.9	376.1	265.7	795.7	439.6	172.8	1023.6	498.3	321.4
Bias SD	743.3	500.5	339.1	966.2	523.7	216.3	1344.2	613.1	413.8
SEM (% on mean)	8.9	6.2	8.8	8.0	9.6	9.6	5.8	4.7	6.8
ICC(2,1)	0.9	0.8	0.9	0.7	0.8	0.9	0.8	0.8	0.9

**Table 2:** AUC ID [×10^7^] and Lag Time (s) parameters in healthy donors. Values shown as mean ± SD, median, 5th–95th percentile, and 95% Confidence Interval (CI). For Lag Time, only mean ± SD is shown. Data derived from 62 donor samples analyzed under identical conditions.

Parameter	Metric	Platelets	Fibrin(ogen)	Total Thrombus
AUC ID [×10^7^]	Mean ± SD	3.6 ± 1.0	5.1 ± 1.8	8.6 ± 2.4
	Median	3.5	5.0	8.4
	5th–95th	1.9–5.4	2.4–9.0	5.6–14.0
	95% CI	3.3–3.8	4.7–5.5	8.0–9.2
Lag Time (s)	Mean ± SD	NA	241 ± 39	NA

NA: not applicable, as the parameter is not defined for platelets or total thrombus signals.

**Table 3:** Ranges of INR, aPTT ratio, platelet count, and fibrinogen concentration for healthy donors and ECC patient groups (pre-ECC, post-ECC t_0_, and post-ECC ICU). n.a.: data not available by surgical protocol. Platelet and fibrinogen levels showed marked post-ECC decreases consistent with Smart Clot profiles.

Group	*n*	INR (Range)	aPTT Ratio (Range)	Platelets (×10^3^/µL, Range)	Fibrinogen (mg/dL, Range)
Healthy donors	62	0.80–1.20	0.80–1.30	150–400	200–400
Pre-ECC (ASA 75–100 mg/day)	22	1.00–1.41	0.80–1.39	142–358	191–484
Post-ECC t_0_ (5–10 min after protamine 300 IU/kg)	6	n.a.	n.a.	n.a.	n.a.
Post-ECC ICU (~1.5 h after protamine)	22	1.03–1.76	0.76–1.67	72–249	106–305

## Data Availability

The raw data supporting the conclusions of this article will be made available by the authors on request.

## Supplementary Materials

The following supporting information can be downloaded at: https://www.mdpi.com/article/10.3390/bios16020080/s1. Table S1: Model comparison between 5-parameter logistic (5PL) and 5th-degree polynomial fitting (Poly-5) for Smart Clot Integrated Density (ID) curves; Table S2: Statistical comparison of normal versus log-normal distribution of Smart Clot quantitative parameters in healthy donors; Table S3: Quantitative thrombus parameters of anticoagulant-treated patients; Table S4: Quantitative thrombus parameters of antiplatelet-treated patients; Table S5: Quantitative thrombus parameters of patients undergoing Extra Corporeal Circulation (ECC); Video S1: Comparative visualization of thrombus formation under flow in a healthy donor versus a patient under dual antiplatelet therapy (DAPT).

## Abbreviations

The following abbreviations are used in this manuscript:

POC	Point-of-care
ID	Integrated density
AUC ID	Area under the first derivative curve
PLTS	Platelets
FIB	Fibrin(ogen)
TT	Total thrombus
5PL	Richard’s five-parameter logistic equation
RSS	Robust sum of squares
VKA	Vitamin K antagonist
DOAC	Direct oral anticoagulant
ASA	Acetylsalicylic acid
P2Y_12_ inh	P2Y_12_ receptor inhibitor (clopidogrel)
DAPT	Dual antiplatelet therapy
ECC	Extracorporeal circulation
ICU	Intensive care unit
SD	Standard deviation
SEM	Standard error of the mean
ICC(2,1)	Intraclass correlation coefficient (two-way random, single measures, absolute agreement)
IQR	Interquartile range
SW	Shapiro–Wilk test
DP	D’Agostino–Pearson test
CI	Confidence interval
vWF	Von Willebrand factor
TGTs	Thrombin generation tests
VASP	Vasodilator-stimulated phosphoprotein
