# Supplementary material for: Smart Clot: An Automated Point-of-Care Flow Assay for Quantitative Whole-Blood Platelet, Fibrin, and Thrombus Kinetics
Source: Biosensors (Basel). 2026 Jan 28;16(2):80. doi: 10.3390/bios16020080 (PMC12938410; doi:10.3390/bios16020080)
Supplement: Supplementary file 1 [file biosensors-16-00080-s001.zip › biosensors-4089479-supplementary.pdf]

biosensors

MDPI

Supplementary Materials

Smart Clot: An Automated Point-of-Care Flow Assay for Quantitative Whole-Blood Platelet, Fibrin, and Thrombus Kinetics

Alessandro Foladore [1], Simone Lattanzio [1], Ekaterina Baryshnikova [2], Martina Anguissola [2], Elisabetta Lombardi [3], Marco Valvasori [3], Roberto Vettori [3], Francesco Agostini [3], Roberto Tassan Toffola [4], Lidia Rota [1], Marco Ranucci [2,†] and Mario Mazzucato [3,*,†]

1 Sedicidodici s.r.l., 33170 Pordenone, Italy; afoladore@1612medical.com (A.F.); slattanzio@1612medical.com (S.L.); lidiarotavender@gmail.com (L.R.)
2 Department of Cardiothoracic and Vascular Anesthesia and Intensive Care, Istituto di Ricovero e Cura a Carattere Scientifico (IRCCS) Policlinico San Donato, 20097 San Donato Milanese, Italy; ekaterina.baryshnikova@grupposandonato.it (E.B.); martina.anguissola@grupposandonato.it (M.A.); marco.ranucci@grupposandonato.it (M.R.)
3 Stem Cell Unit, Department of Research and Advanced Cancer Diagnostic, Centro di Riferimento Oncologico di Aviano (CRO) IRCCS, 33081 Aviano, Italy; elombardi@cro.it (E.L.); marco.valvasori@cro.it (M.V.); rvettori@cro.it (R.V.); fagostini@cro.it (F.A.)
4 Unit of Transfusion Medicine in Oncology, Department of Transfusion Medicine, Azienda Sanitaria Friuli Occidentale, 33170 Pordenone, Italy; roberto.tassantoffola@asfo.sanita.fvg.it
* Correspondence: moreno.mazzucato@libero.it; Tel.: +39-0434659058
† These authors contributed equally to this work.

Supplementary Table S1. Model comparison between 5-parameter logistic (5PL) and 5th-degree polynomial fitting (Poly-5) for Smart Clot Integrated Density (ID) curves.

A comparison of two fitting models applied to the mean Integrated Density (ID) time-series from healthy donors (n = 62). The 5-parameter logistic (5PL) model showed lower Robust Sum of Squares (RSS) than the 5th-degree polynomial (Poly-5) for all analytical components, indicating superior fit quality and smoother derivative behavior. A positive ΔRSS (Poly − 5PL) corresponds to a better fit of the 5PL model. Values are expressed as mean ± standard deviation (SD); ΔRSS > 0 (%) indicates the percentage of curves where the 5PL model outperformed the polynomial model.

Parameter	Mean RSS ± SD (5PL)	Mean RSS ± SD (Poly-5)	ΔRSS (Poly − 5PL) Mean ± SD	ΔRSS > 0 (%)
Platelets	130 ± 40	212 ± 64	+105 ± 81	94
Fibrin(ogen)	150 ± 71	223 ± 73	+105 ± 86	89
Total Thrombus	129 ± 38	223 ± 66	+112 ± 79	90

Supplementary Table S2: Statistical comparison of normal versus log-normal distribution of Smart Clot quantitative parameters in healthy donors.

The results of Shapiro–Wilk and D'Agostino–Pearson tests applied to platelets, fibrin(ogen), and total thrombus mean area under the first derivative curve (AUC ID) values. For platelets, *p*-values were > 0.05 for both normality and log-normality tests, indicating that neither distribution could be rejected. In contrast, fibrin(ogen) and total thrombus values showed $p < 0.05$ for normality but $p > 0.05$ for log-normality, supporting a right-skewed log-normal pattern. These results are consistent with the QQ plots shown in Figure 2.

Parameter	Shapiro–Wilk *p* (normal)	Shapiro–Wilk *p* (log-normal)	D'Agostino–Pearson *p* (normal)	D'Agostino–Pearson *p* (log-normal)	Final Interpretation
Platelets	0.722	0.658	0.391	0.333	Both acceptable
Fibrin(ogen)	0.036	0.725	0.012	0.352	Log-normal distribution accepted
Total Thrombus	0.037	0.896	0.030	0.951	Log-normal distribution accepted

Supplementary Table S3: Quantitative thrombus parameters of anticoagulant-treated patients.

Mean area under the curve (AUC ID) from derivative-based kinetic analysis in patients on direct oral anticoagulants (DOACs, n = 18) and vitamin K antagonists (VKAs, divided by INR, n = 6+6). Values are shown as mean ± standard deviation (SD), median, 5th–95th percentile, and 95% confidence interval (CI). INR ranges for VKA-treated patients are reported in the last row.

Parameter	Metric	DOACs (*n*=18)	VKA INR <2 (*n*=6)	VKA INR >2 (*n*=6)
Platelets AUC ID [x10^7]	Mean ± SD	1.53 ± 0.67	1.89 ± 1.01	1.11 ± 0.82
	Median	1.38	1.65	0.90
	5th–95th	0.62–2.67	0.40–3.15	0.23–2.52
	95% CI	1.19–1.86	0.84–2.95	0.25–1.97
Fibrin(ogen) AUC ID [x10^7]	Mean ± SD	1.90 ± 1.15	3.00 ± 1.95	0.82 ± 0.54
	Median	1.84	3.04	0.85
	5th–95th	0.39–4.40	0.03–5.54	0.09–1.53
	95% CI	1.32–2.47	0.95–5.05	0.25–1.38
Total Thrombus AUC ID [x10^7]	Mean ± SD	3.35 ± 1.49	4.75 ± 2.52	1.87 ± 1.23
	Median	3.46	5.07	1.73
	5th–95th	1.20–5.74	0.44–7.87	0.38–3.79
	95% CI	2.60–4.09	2.10–7.39	0.59–3.16
INR range		n.a.	1.27–1.94	2.01–3.21

Supplementary Table S4: Quantitative thrombus parameters of antiplatelet-treated patients.

Mean area under the curve (AUC ID) from derivative-based kinetic analysis in patients treated with acetylsalicylic acid (ASA, *n* = 24), clopidogrel (P2Y12 inh, *n* = 11) and dual antiplatelet therapy (DAPT: ASA + clopidogrel, *n* = 32). Values are shown as mean ± standard deviation (SD), median, 5th–95th percentile, and 95% confidence interval (CI).

Parameter	Metric	ASA (*n*=24)	$P2Y_{12}$ inh (*n*=11)	DAPT (*n*=32)
Platelets AUC ID [x10^7]	Mean ± SD	2.88 ± 1.15	1.61 ± 0.89	1.17 ± 0.56
	Median	2.59	1.40	1.20
	5th–95th	1.53–5.63	0.59–3.76	0.22–2.55
	95% CI	2.40–3.36	1.01–2.21	0.97–1.37
Fibrin(ogen) AUC ID [x10^7]	Mean ± SD	2.89 ± 1.10	1.97 ± 1.41	1.26 ± 0.67
	Median	2.94	1.52	1.35
	5th–95th	0.94–4.96	0.32–5.60	0.99–2.32
	95% CI	2.42–3.35	1.02–2.91	1.02–1.50
Total Thrombus AUC ID [x10^7]	Mean ± SD	5.80 ± 1.94	3.57 ± 2.19	2.41 ± 1.00
	Median	5.87	2.94	2.53
	5th–95th	2.96–9.57	0.91–8.62	0.47–3.92
	95% CI	4.98–6.62	2.10–5.04	2.05–2.77

Supplementary Table S5. Quantitative thrombus parameters of patients undergoing Extra Corporeal Circulation (ECC)

Mean area under the curve (AUC ID) from derivative-based kinetic analysis in pre-extracorporeal circulation (ECC) patients (*n* = 22), post-ECC t_0 (*n* = 6, 5–10 min after protamine 300 IU/kg), and post-ECC ICU samples (*n* = 22, ≈1.5 h after protamine). Values are shown as mean ± standard deviation (SD), median, 5th–95th percentile, and 95% confidence interval (CI).

Parameter	Metric	Pre-ECC (*n*=22)	Post-ECC (t_0) (*n*=6)	Post-ECC ICU (*n*=22)
Platelets AUC ID [x10^7]	Mean ± SD	3.07 ± 1.52	0.39 ± 0.55	1.13 ± 0.81
	Median	2.53	0.19	0.94
	5th–95th	1.14–6.71	0.12–1.49	0.22–2.95
	95% CI	2.39-3.74	-0.18–0.95	0.77–1.49
Fibrin(ogen) AUC ID [x10^7]	Mean ± SD	3.32 ± 1.43	0.27 ± 0.23	1.04 ± 0.84
	Median	2.95	0.19	0.95
	5th–95th	1.03–6.44	0.15–0.74	0.06–2.66
	95% CI	2.68–3.95	0.03–0.51	0.67–1.41
Total Thrombus AUC ID [x10^7]	Mean ± SD	6.36 ± 2.66	0.64 ± 0.75	2.11 ± 1.60
	Median	5.85	0.34	2.08
	5th–95th	2.04–12.8	0.29–2.16	0.26–5.55
	95% CI	5.18–7.54	-0.14–1.42	1.40–2.82

Supplementary Video S1: Comparative visualization of thrombus formation under flow in a healthy donor versus a patient under dual antiplatelet therapy (DAPT).

The movie shows, on the left, a blood sample from a healthy donor (control) and, on the right, a patient treated with dual antiplatelet therapy (acetylsalicylic acid 100 mg/day + clopidogrel 75 mg/day). Whole blood was recalcified and tested under a shear rate of 300 s^{-1}, reproducing physiological arterial flow. Images were recorded at 1 frame/s and the final sequence was edited at 7 frames/s for faster visualization. Platelets are labeled in green ($DiOC_6(3)$) and fibrin(ogen) in red (Alexa Fluor 546). Fibrinogen appears as punctate structures between platelets, whereas fibrin—formed through thrombin cleavage—appears as interlaced mesh fibers, defining the 3D scaffold of the forming thrombus. Smart Clot clearly detects the marked suppression of platelet aggregation and fibrin network formation in DAPT-treated blood compared to the healthy donor. This video visually complements Figure 4 of the main manuscript.
